# Client Oriented Scale of Improvement in First-Time and Experienced Hearing Aid Users: An Analysis of Five Predetermined Predictability Categories through Audiometric and Speech Testing

**DOI:** 10.3390/jcm13133956

**Published:** 2024-07-05

**Authors:** Pietro Salvago, Davide Vaccaro, Fulvio Plescia, Rossana Vitale, Luigi Cirrincione, Lucrezia Evola, Francesco Martines

**Affiliations:** 1 Dipartimento di Biomedicina, Neuroscienze e Diagnostica Avanzata (BiND), Sezione di Audiologia, Università degli Studi di Palermo, Via del Vespro 129, 90127 Palermo, Italy; francesco.martines@unipa.it; 2 UOSD Audiologia, Azienda Ospedaliera Universitaria Policlinico—A.O.U.P. “Paolo Giaccone”, Via del Vespro 129, 90127 Palermo, Italy; davide.vaccaro1993@libero.it (D.V.); rossana.vitale1995@gmail.com (R.V.); lucrezia.evola@hotmail.it (L.E.); 3 Dipartimento di Promozione della Salute, Materno-Infantile, di Medicina Interna e Specialistica di Eccellenza “G. D’Alessandro”, University of Palermo, Via del Vespro 133, 90127 Palermo, Italy; fulvio.plescia@unipa.it (F.P.); luigi.cirrincione@unipa.it (L.C.)

**Keywords:** hearing aids, client-oriented scale of improvement, hearing aid users, speech audiometry

## Abstract

**Objectives:** The aim of our investigation was to explore the relationship between unaided pure-tone and speech audiometry and self-reported aided performance measured according to five predetermined COSI categories among first-time hearing aid users and experienced hearing aid users. **Methods:** Data from 286 patients were retrospectively evaluated. We divided the sample into first-time hearing aid users (G1) and experienced hearing aid users (G2). The correlation between unaided tonal and speech audiometry and five preliminary selected client-oriented scale of improvement (COSI) categories was studied. **Results:** A greater percentage of hearing aid users aged >80 years and a higher prevalence of severe-to-profound hearing loss in G2 group were observed (*p* < 0.05). For the total cohort, a mean hearing threshold of 60.37 ± 18.77 db HL emerged in the right ear, and 59.97 ± 18.76 db HL was detected in the left ear (*p* > 0.05). A significant statistical difference was observed in the group of first-time hearing aid users for the “Television/Radio at normal volume” item, where patients with a lower speech intellection threshold (SIT) were associated with higher COSI scores (*p* = 0.019). Studying the relationship between the speech reception threshold (SRT) and the COSI item “conversation with 1 or 2 in noise” evidenced worse speech audiometry in patients who scored ≤2 among experienced hearing aid users (*p* = 0.00012); a higher mean 4–8 kHz frequencies threshold for the better ear was found within the G2 group among those who scored ≤2 in the COSI item “conversation with 1 or 2 in quiet” (*p* = 0.043). **Conclusions:** Our study confirms a poor correlation between unaided tonal and speech audiometry and self-reported patient assessment. Although we included only five COSI categories in this study, it is clear that unaided audiometric tests may drive the choice of proper hearing rehabilitation, but their value in predicting the benefit of hearing aids remains limited.

## 1. Introduction

Recognized as one of the major causes of disability, hearing loss affects 1.5 billion people around the world, with an increasing prevalence as the population ages [1]. In fact, it was estimated that more than 30% of people aged >65 years old suffer from hearing loss, while an 80% prevalence rate may be found among those aged >80 years old [2]. Presbycusis represents the most common cause of hearing loss; this condition, also known as “age-related hearing loss” (ARHL), is a multifactorial disease where heredity, noise exposure, stress, drugs, metabolic, and vascular conditions may all play a role in its development [3,4]. 

When untreated, hearing loss may result in poor speech discrimination, which, in turn, may lead to communication difficulties, social isolation, depression, and a reduced quality of life [5]. Modern hearing aids, which detect, elaborate, and amplify sounds, are the primary clinical intervention; however, the use of hearing aids to amplify sounds does not necessarily restore hearing function [6,7]. In fact, frequency response characteristics of hearing aids, distortions arising from peak clipping, and poor clarity or loudness of speech could all have an impact on successful listening, rendering the patient unsatisfied, particularly in challenging listening situations (e.g., noisy backgrounds) [8,9].

Audiological tests are commonly used in the selection process of hearing aid candidates as well as in objectively measuring functional gain, speech recognition improvement, and the real-ear responses provided when wearing a hearing aid [10,11]. However, these measurements partially reflect the benefits of hearing in real-life situations and demonstrate poor predictability of the subjective difficulty experienced during daily listening situations [12].

A useful tool for assessing the benefits of hearing aids still remains the use of questionnaires, which are administered to patients in order to rate the improvement of listening in different environments as well as to investigate psychological and emotional aspects before and after hearing rehabilitation [13,14].

Some popular instruments for measuring the benefits of hearing aids include the abbreviated profile of hearing aid benefit [15], the speech, spatial, and qualities of hearing scale (SSQ) [16], and the client-oriented scale of improvement (COSI) [17]. This latter instrument permits the patient to choose up to five situations where they would like to cope better; in so doing, the evaluation of the patient is better centred on their personal needs and gives the clinician a more realistic picture of situations which are relevant for the listeners, which should be targeted when fitting hearing aids. However, its applicability for research purposes is limited as it complicates the comparison of hearing-aid-related benefits across patients. To improve comparability between patients, Dillon et al. proposed designating each individual target into a set of 16 predefined categories [17].

Even though audiometry, speech perception testing, and questionnaires may suggest different aspects of the outcome of hearing aid rehabilitation to the clinician, authors have previously focused on their interaction in order to find correlation between patients’ characteristics, objective measures, and subjective aided experience. For example, Lansbergen et al. did not find any significant correlation between pure-tone audiometry in the better hear and COSI final ability [18]. Similarly, when assessing the COSI responses of a non-sampled population, Windle did not find evidence of any significant associations between hearing aid benefits and age or degree of hearing loss [19]. Dornhoffer et al. showed that correlations between audiologic and patient-reported measures of aided performance or hearing aid benefit were low-to-weak or absent when examining unaided and aided data from the preoperative profiles of 95 experienced hearing aid users [20]. In contrast, Suresh et al. revealed that one of the most important features in predicting hearing aid improvement was a higher pure-tone average in the better hearing ear in their study on developing a model with which to predict individualized hearing aid benefits [21].

The main goal of this study is to explore the relationship between audiologic tests, including pure-tone and speech audiometry, and self-reported aided performance measured according to five predetermined COSI items; in particular, the research aims to investigate whether there are any predictors of COSI outcomes among first-time hearing aid users and experienced hearing aid users.

## 2. Materials and Methods

### 2.1. Subjects

To address the objectives of this study, we retrospectively collected data from a total of 286 hearing aid users who were recruited from the Department of Audiology at the Policlinico “P. Giaccone” University Hospital of Palermo (Italy) between January 2022 and June 2023.

Exclusion criteria included the following: patients aged <18 years, pre-lingual deafness, bimodal users, unilateral hearing loss, severe neurologic and psychiatric disorders, and uncooperative patients. 

We recruited 140 women and 146 men (male–female ratio = 1.04), and the total cohort presented a mean age of 74 ± 11.57 years of age. All participants suffered from bilateral sensorineural hearing loss (SNHL) and/or mixed hearing loss and were fitted with digital, multichannel compression hearing aids. Hearing aid counseling, fitting, verification, and validation were all performed by the same hearing aid dispenser according to standard procedure. The prescription formula used was the nonlinear fitting procedure version 1 (NAL-NL1) of the National Acoustic Laboratories. Patients were divided into two groups: first-time hearing aid users (G1) and experienced hearing aid users (G2). 

The Ethics Committee decided that no ethical approval statement was required for this study, considering the absence of invasive procedure, lack of randomization, and the patient’s conscious choice of hearing aid.

### 2.2. Audiometric and Speech Assessment

After careful anamnesis, patients underwent micro-otoscopy to rule out middle-ear pathologies and/or active infections. Pure-tone audiometry (PTA) was performed by a trained audiologist with a Piano clinical audiometer (Inventis, Padua, Italy) in a soundproof audiometric room. Air conduction was measured without hearing aids using an on-ear TDH-49 headphones set to 250–8000 Hz; bone conduction was measured using a calibrated bone transducer at 250–4000 Hz.

To ensure the stability of middle ear function, tympanometry was performed using a Clarinet clinical tympanometer (Inventis, Padua, Italy) with a probe frequency of 226 Hz and an air pressure range of −400 to +200 daPa with automatic recording.

Speech recognition was studied with a Piano clinical audiometer (Inventis, Padua, Italy) under quiet conditions in a soundproof booth, and tests were carried out by experienced audiologists using standard clinical protocols. Speech recognition in quiet conditions was administered using an open-set, phonemically balanced word test. Ten meaningful disyllabic words were presented using on-ear TDH-49 headphones at different intensities. Listeners were instructed to repeat each word. The percentage of correct answers (word recognition score, WRS) was calculated to determine a score of three different levels of speech recognition: (1) the minimum hearing level (dB HL) for speech at which an individual can just detect the presence of speech material 0% of the time (speech detection threshold, SDT); (2) the minimum hearing level (dB HL) for speech at which an individual can recognise 50% of the speech material (speech reception threshold, SRT); (3) the lowest intensity (dB HL) at which the patient can recognise 100% of words, also referred to as the “Speech Intellection Threshold” (SIT) in Italy.

### 2.3. Client-Oriented Scale of Improvement (COSI)

The client-oriented scale of improvement (COSI) is a clinical tool developed by the National Acoustic Laboratories (NAL) for outcome measurement [17]. It is an assessment questionnaire for clinicians to use which allows them to document their client’s goals/needs and measures improvements in hearing ability.

To use the COSI, patients identify up to five specific situations that they would like to improve by wearing amplification. These situations can be listening situations or they can be emotional or social situations. Next, the audiologist records the appropriate standard category on the COSI form for each of the specific situations. Patients are asked to rate the degree of change in hearing ability for each specific listening situation on a 5-point scale: 1 for “worse”, 2 for “no difference”, 3 for “slightly better”, 4 for “better”, and 5 for “much better”.

During the first appointment patients were asked to identify three primary situations in which they would like to improve their hearing. To better address the goals of our research, we preliminarily decided to include only 5 COSI categories in the study, which were the result of the most-requested listening situations in which the sample patients would prefer to improve: (1) television/radio at normal volume; (2) conversation with 1 or 2 people in quiet; (3) conversation with 1 or 2 people in noise; (4) church or meeting; (5) feeling left out. Then, 6 months after they were provided with hearing aids, they were assessed using the 5 COSI predetermined items. 

Additionally, we asked patients to rate the change in their ability to localize sounds on a five-point scale, even if the item was not included in the COSI questionnaire categories. 

### 2.4. Statistical Analysis

Continuous variables were represented as mean ± standard deviation (SD), and categorical variables were represented as a number and a percentage. Data normality was assessed using the Kolmogorov–Smirnov normality test. Comparisons between categorical variables were performed using the chi-square test and Fisher’s exact test. The Mann–Whitney U test was used to analyse continuous variables without a normal distribution. Spearman’s correlation coefficients were also calculated. A univariate linear regression analysis was used to assess the possible relationships between audiometry, speech testing, and COSI outcomes. A two-tailed *p* < 0.05 was considered significant. The STATISTICA Software, Palermo, Italy (Vers. 8.0 for Windows) was utilized.

## 3. Results

Table 1 summarizes the main demographic and audiological features of the whole sample studied. A total of 113 patients, 54 women and 59 men, were first-time hearing aid users (G1), while 173 patients, 86 women and 87 men, were experienced hearing aid users (G2); no significant difference was found in the distribution of sex between groups (*p* = 0.75). The mean age of patients was 74.27 ± 8.74 years and 73.82 ± 13.11 years in the G1 and G2 groups, respectively (*p* = 0.74); a greater percentage of hearing aid users aged >80 years (29.43%) was observed in the G2 group with respect to the G1 group (18.58%), with a statistically significant difference (*p* = 0.009).

**Figure 1:**
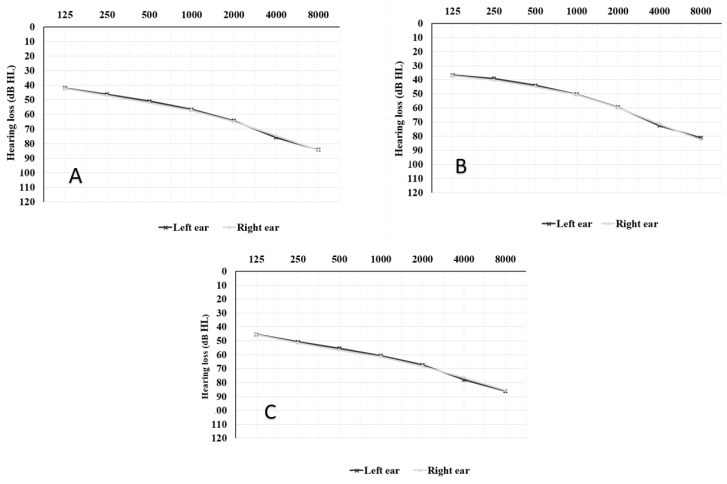
Mean audiogram of the total cohort (**A**), G1 (**B**), and G2 (**C**) groups.

The study of mean pure-tone audiometry (PTA250-8000 Hz) in the total cohort (Figure 1) showed a mean hearing threshold of 60.37 ± 18.77 db HL in the right ear and 59.97 ± 18.76 db HL in the left ear (*p* > 0.05), with a high-frequency gently sloping audiogram; specifically, there was a mean PTA250-8000 Hz of 55.30 ± 16.46 db HL in the right ear and 54.77 ± 16.31 db HL in the left ear of the G1 group, while a mean hearing threshold of 63.69 ± 19.37 db HL in the right ear and 63.38 ± 19.44 db HL in the left ear was found in G2 patients (*p* > 0.05). No significant differences were found in the mean hearing thresholds of the left and right ears within groups (*p* > 0.05). Both mean audiograms (Figure 1B,C) presented a gently sloping shape, with G2 patients presenting a mean 10 db HL audiogram that was worse for each frequency explored. Analysis of the degree of hearing loss among groups revealed a higher prevalence of severe-to-profound hearing loss in the G2 group with respect to the G1 group. In particular, 32.36% of experienced hearing aid users and 12.83% of first-time hearing aid users suffered from severe-to-profound hearing loss in at least one ear (*p* = 0.00001). An SNHL was diagnosed in most cases (86.53%), without any significant difference in the prevalence of mixed hearing loss between G1 and G2 groups (*p* = 0.54); no cases of conductive hearing loss were found. 

Speech audiometry testing demonstrated a significantly higher SDT and SRT in the G2 group with respect to the G1 group (*p* < 0.05); with regard to SIT, a significant difference was observed only in the left ear, with a mean value of 72.04 db HL in first-time hearing aid users and of 80 db HL in experienced hearing aid users. In addition, 86.53% of ears in the G2 group did not reach the SIT (*p* = 0.00001); only 3.09% of G1 ears and 4.04% of G2 ears presented a word recognition lower than 50% at the maximum level tested (*p* = 0.55).

**Figure 2:**
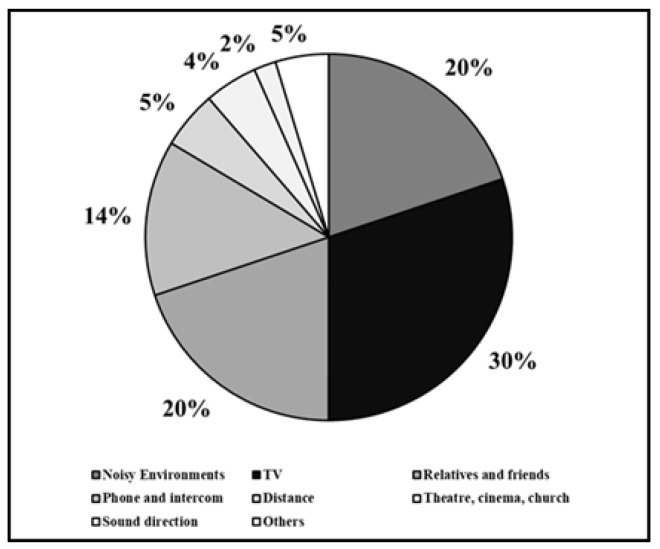
Main acoustic situations in which patients would like to improve their hearing with hearing aids.

As shown in Figure 2, the four main acoustic situations in which patients would like to improve their hearing were listening to TV (30.15%), hearing in noisy environments (19.84%), having a conversation with relatives and friends (19.84%), and hearing the phone and intercom (13.7%).

After assessing patients with the COSI questionnaire, a better outcome in first-time hearing aid users emerged in the following listening situations: attending church or meetings (3.05 ± 0.61, *p* = 0.15) and feeling left out (3 ± 0.75, *p* = 0.00001), with a statistically significant difference only for the latter situation; in addition, G1 patients rated improvement in the ability to localize sounds higher than experienced hearing aid users (3.39 ± 1, *p* = 0.03). No significant difference was found in case of “Television/Radio at normal volume” (*p* = 0.96) and “Conversation with 1 or 2 in noise” (*p* = 0.92) items between first-time and experienced hearing aid users.

**Figure 3:**
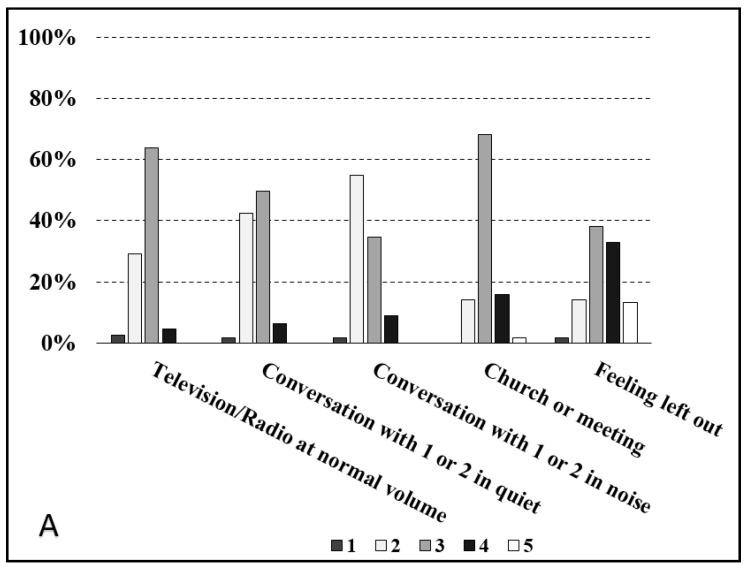

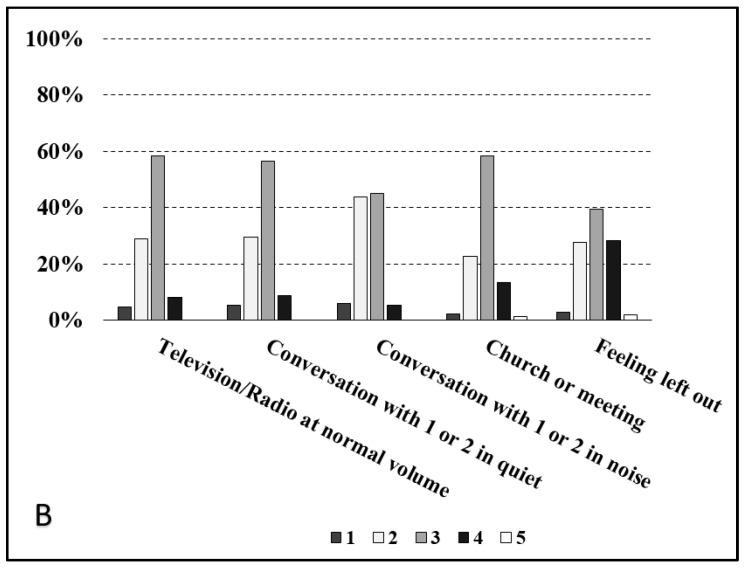
Distribution of COSI outcomes among G1 (**A**) and G2 (**B**) groups.

With a score ≥4, better outcomes were observed for “feeling left out” in both groups, while the lowest prevalence of high scores was found for “Television/Radio at normal volume” in the G1 group and conversation with one or two people in the noise item in the G2 group (Figure 3); however, no significant difference was recognized in terms of age between those who scored ≥4 and those who scored ≤3 on the question regarding feeling left out.

From studying the relationship between SRT and the COSI item “conversation with 1 or 2 in noise”, a worse speech audiometry was identified in patients who scored ≤2 among experienced hearing aid users (*p* = 0.00012). A significant statistical difference was also found among first-time hearing aid users for “Television/Radio at normal volume”, with patients with a lower SIT being associated with higher COSI scores (*p* = 0.019).

No correlation was found between age and the hearing threshold in both groups, while a weak positive correlation was observed between age and SRT in the G1 group (rs = 0.29). Studying the relationship between audiometry and COSI, we identified a significant weak negative correlation in the G2 group for all items except “feeling left out” (rs = −0.13, *p* = 0.08); the hearing threshold showed a weak negative correlation with “Television/Radio at normal volume” (rs = −0.21, *p* = 0.02) and “Church or meetings” (rs = −0.33, *p* = 0.0003) in the G1 group.

Finally, a significant difference in the mean 4–8 kHz frequencies threshold in the better ear was found within the G2 group between those who scored ≤2 and those who expressed a better outcome in the COSI item “conversation with 1 or 2 in quiet” (*p* = 0.043).

## 4. Discussion

Apart from determining a patient’s hearing ability, audiological tests may help clinicians select patients who may benefit from hearing amplification [11]; unfortunately, even if different audiometric and speech tests are available, they do not fully reflect the acoustic environments faced by hearing-impaired patients in everyday life [18]. Previous studies have demonstrated the low predictability of pure-tone audiometry for subjective satisfaction of people with hearing aids, particularly when the benefit of amplification was assessed through standardized questionnaires [22,23,24]. This lack of correlation between audiometric tests and patient-reported outcome measures can be viewed in light of the numerous epidemiological, psychological, and technological variables that might interfere with the rehabilitation process and that are still a focus of current research. 

Our study focused on two different subgroups of hearing aid users since the duration of amplification may influence results in the long term, driven by the acclimatization effect and neurophysiological-related changes [25,26,27]. To begin with, experienced hearing aid users proved to be older, with a more severe degree of hearing loss and a lower speech recognition outcome than first-time hearing aid users (*p* < 0.05). Interestingly, the latter group reported better results at the COSI question regarding “feeling left out” (*p* = 0.00001) and in the localization of sounds (*p* = 0.03). The most likely explanation is that first-time hearing aid users may immediately perceive the benefits of amplification and once again feel comfortable in social life. Instead, patients from the G2 group were more susceptible to depression and anxiety because of their older age; nevertheless, this group showed the highest COSI results on the “feeling left out” question with respect to the other items. On the other hand, the higher hearing threshold of G1 patients may help them to localize sounds better than experienced hearing aid users. 

Even if the worst mean outcome was found for “conversation with 1 or 2 in noise” (2.49 ± 0.7), the G1 group showed the lowest prevalence of high scores (4–5) for “Television/Radio at normal volume”, with this item being predicted by SIT values (*p* = 0.019). However, this result can also be viewed in light of speech recognition ability because other factors such as the use of connectivity devices may influence the quality of TV listening. Experienced hearing aid users presented the worst outcomes on the “conversation with 1 or 2 in noise” question. In particular, a lower SRT among those who scored ≤2 within this group was the result. The ability to distinguish words and or sentences from background noise has always represented the most challenging acoustic situation for hearing aid listeners. This ability, particularly in noisy environments, tends to decline with age, with a mean deterioration in speech recognition in noisy conditions of 1.37 and 1.69 dB SNR over 10 years among 51–60 and 61–70 year-old patients, respectively (corresponding to a 27% and 34% decrease in speech understanding) [28]. Speech perception requires a listener to find an acceptable match between the incoming acoustic signal generated by the talker and a linguistic structure likely to be similar to the one intended by the talker. The process of speech perception appears to be automatic and effortless every time the sensory input provided by a talker is audible and uncorrupted with a message that is not unexpected or cognitively difficult. The corruption of sensory input may slow the process of speech recognition, increasing dependence on inferences based on partial sensory information, the listener’s knowledge and understanding of the topic, and other communication circumstances. The greater the corruption of the speech signal, the greater the effort needed to arrive at a plausible hypothesis about the message [29].

Even when wearing hearing aids, patients suffering from SNHL do not acquire complete information from the acoustic signal, at least not to the extent of the redundant information usually available to normal-hearing people. Missed information may be filled in through top-down pathways, requiring more listening effort from hearing-impaired listeners [30]. Cognitive resources, such as working memory capacity, may partially account for individual differences in speech perception outcomes between hearing aid users with similar pure-tone thresholds [31]. 

Various investigations have reported an association between cognitive abilities and speech perception outcomes. Gatehouse et al. found that better cognitive ability in experienced hearing aid users, measured using visual digit and letter-monitoring tasks, was associated with better speech recognition in noisy conditions [32]. Lunner et al. also observed better speech recognition performance in both aided and unaided conditions among hearing aid users with good cognitive abilities [33]. Other authors have reported that speech perception performance may be influenced by general processing speed, lexical access speed, and phonological processing skills [30,33,34]. In particular, degraded phonological representation, which is commonly found in patients with severe hearing impairment, may make the process of listening in the presence of background noise challenging. In fact, when the incoming speech signal is masked by noise, a mismatch condition between the incoming signal and the phonological representation may arise. This condition, from distortion of the incoming signal due to cochlear damage, may create an important source of mismatch, which may explain why SNHL patients can experience disproportionate difficulty listening in the presence of background noise. Hearing aids may generate further side-effects, such as generating unwanted artifacts in the auditory scene or distorting the waveform of the speech signal [35,36]. Consequently, the new hearing aid user may not find congruency between aided incoming signals and nonaided phonological representations in long-term memory. After becoming accustomed to hearing aid amplification and settings, the degree of mismatch may be reduced as new phonological representations that are congruent with the processed speech input may become established in the lexicon over time [37]. 

In our study, unaided audiometry was found to be a poor predictor of patient-reported outcomes, showing only a weak negative correlation in the G2 group for all items (*p* < 0.05) except for “feeling left out” and in the G1 group for “Television/Radio at normal volume” (*p* = 0.02) and “Church or meetings” (*p* = 0.0003). Specifically, among experienced hearing aid users, a lower 4–8 kHz threshold in the better ear was related to better performance when conversing in quiet situations (*p* = 0.043). This was not surprising because a good 4–8 kHz threshold in one ear helps speech discrimination in quiet situations, but only good binaural function allows the patient to understand conversation in the context of background noise. Previous studies show similar outcomes to the current study, with absent to low correlations between patient-reported outcomes [23,24,38]. In a multiple regression analysis. Chang et al. reported a significant but small negative association of the unaided word recognition score with the hearing handicap inventory for the elderly at one month but a weak positive correlation at 3 months [39]; other authors demonstrated no correlation in a similar sample, as well as in a large meta-analysis [40,41].

Our study presents the following limitations. To begin with, we reported audiometric and speech tests without wearing hearing aids; we are aware that tonal and speech audiometry under aided conditions might be better related to patient-reported outcomes, but data are still controversial, with studies reporting low-to absent correlations even when considering aided measures [20,42,43]. In addition, the retrospective nature of the study and the single-center setting might affect the generalizability of the findings. Secondly, more variables such as duration of hearing aid amplification, patient demographics, and cognitive functions may have influenced COSI outcomes among hearing aid users. Finally, to render data comparable between patients, we opted to identify the most common acoustic situations preliminarily (unconventionally using five fixed COSI categories) in which hearing-impaired patients wished to improve their hearing. We are aware that this may not reflect the individual needs of rehabilitation of the total sample, but they might be considered common acoustic complaints, and for this reason, they were believed to be better adapted to interindividual comparison.

## 5. Conclusions

Our study confirms a poor correlation between unaided tonal and speech audiometry and patient self-reported assessments. Even if first-time and experienced hearing aid users represent two distinct populations, few significant predictors of COSI outcomes were found; in particular, SIT was found to be associated with low scores of “Television/Radio at normal volume” in first-time hearing aid users, and SRT was found to have worse outcomes for the “conversation with 1 or 2 in noise” item among experienced hearing aid users. Although we included only five COSI categories in the study, it is clear that unaided audiometric tests may drive the choice of proper hearing rehabilitation, but their value at predicting the benefits of hearing aids remains limited by the numerous variables that influence amplification success. 

## Figures and Tables

**Table 1:** Demographic and audiological characteristics of first-time (G1) and experienced hearing aid users (G2).

	Total Cohort N (%)	First Time HA Users (G1) N (%)	Experienced HA Users (G2) N (%)	Statistical Analysis (*p*)
Sex				0.75
Male	140 (48.95)	54 (47.78)	86 (49.71)	
Female	146 (51.05)	59 (52.22)	87 (50.29)	
Age				0.009
≤60 years	31 (10.85)	7 (6.2)	24 (13.89)	
61–79 years	155 (54.19)	73 (64.6)	82 (47.39)	
≥80 years	100 (34.96)	33 (29.20)	67 (38.72)	
Hearing loss type				0.54
Sensorineural	495 (86.53)	198 (87.61)	297 (85.83)	
Mixed	77 (13.47)	28 (12.39)	49 (14.17)	
Hearing loss degree				0.00001
Mild	34 (5.94)	22 (9.73)	14 (4.05)	
Medium	397 (69.4)	182 (80.53)	225 (65.03)	
Severe	110 (19.23)	19 (8.41)	90 (26.01)	
Profound	31 (5.43)	3 (1.33)	17 (4.91)	
Mean PTA (dB HL)				
Left	59.97 ± 18.76	54.77 ± 16.31	63.38 ± 19.44	0.15
Right	60.37 ± 18.77	55.30 ± 16.46	63.69 ± 19.37	0.14
SDT (dB HL)				
Left	47.30 ± 15.25	43.18 ± 14.83	50.05 ± 14.95	0.00012
Right	49.48 ± 16.28	44.24 ± 16.31	52.95 ± 15.41	0.00001
SRT (dB HL)				
Left	64.78 ± 14.63	60.98 ± 12.95	67.32 ± 15.1	0.0003
Right	64.05 ± 13.32	61.11 ± 13.19	65.97 ± 13.1	0.003
SIT (dB HL)				
Left	74.65 ± 13.15	72.04 ± 12.24	80 ± 13.59	0.014
Right	72.77 ± 11.22	71.77 ± 10.93	74.79 ± 11.74	0.57

Abbreviations: PTA: pure-tone audiometry; SDT: speech detection threshold; SRT: speech reception threshold; SIT: speech intellection threshold.

## Data Availability

The data presented in this study are available on request from the corresponding author. The data are not publicly available due to privacy restrictions.
